# Sox10 Controls Migration of B16F10 Melanoma Cells through Multiple Regulatory Target Genes

**DOI:** 10.1371/journal.pone.0031477

**Published:** 2012-02-21

**Authors:** Ikjoo Seong, Hyun Jung Min, Jung-Hyun Lee, Chang-Yeol Yeo, Dong Min Kang, Eok-Soo Oh, Eun Sook Hwang, Jaesang Kim

**Affiliations:** Division of Life and Pharmaceutical Sciences, Ewha Womans University, Seoul, Korea; Univesity of Texas Southwestern Medical Center at Dallas, United States of America

## Abstract

It is believed that the inherent differentiation program of melanocytes during embryogenesis predisposes melanoma cells to high frequency of metastasis. Sox10, a transcription factor expressed in neural crest stem cells and a subset of progeny lineages, plays a key role in the development of melanocytes. We show that B16F10 melanoma cells transfected with siRNAs specific for Sox10 display reduced migratory activity which in turn indicated that a subset of transcriptional regulatory target genes of Sox10 is likely to be involved in migration and metastasis of melanoma cells. We carried out a microarray-based gene expression profiling using a Sox10-specific siRNA to identify relevant regulatory targets and found that multiple genes including melanocortin-1 receptor (Mc1r) partake in the regulation of migration. We provide evidences that the effect of Sox10 on migration is mediated in large part by Mitf, a transcription factor downstream to Sox10. Among the mouse melanoma cell lines examined, however, only B16F10 showed robust down-regulation of Sox10 and inhibition of cell migration indicating that further dissection of dosage effects and/or cell line-specific regulatory networks is necessary. The involvement of Mc1r in migration was studied in detail in vivo using a murine metastasis model. Specifically, B16F10 melanoma cells treated with a specific siRNA showed reduced tendency in metastasizing to and colonizing the lung after being injected in the tail vein. These data reveal a cadre of novel regulators and mediators involved in migration and metastasis of melanoma cells that represents potential targets of therapeutic intervention.

## Introduction

Melanocytes originate from the neural crest cells during embryonic development [1], [2]. Like other cell types from the same lineage, developing melanocytes undergo an extensive migration prior to fully differentiating into pigment producing cells of epidermis and hair follicles. Melanoma cells are malignant cancer cells of melanocytes. Highly metastatic, primary melanomas localized to epidermis are normally not life-threatening, but no efficient treatments exist post metastatic conversion [2], [3]. It has been shown that the very differentiation program that dictates migration during embryogenesis predisposes melanoma cells to metastasis [2], [4]. In particular, Weinberg and co-workers compared the metastatic behavior of melanocytes, fibroblasts, and epithelial cells after introducing identical set of transforming genes to each cell type and found that melanocytes attain metastatic characteristics by far the most efficiently [4]. This implies that a set of lineage specific factors expressed in melanocytes but not in others are at least partly attributable for the metastatic proclivity of this cell type.

Sox10 is a transcription factor belonging to the HMG-box transcription factor family expressed in neural crest stem cells and a subset of derivative lineages including melanocytes [5], [6]. In addition to its role as a multipotency factor in stem cells, Sox10 has been implicated in expression of lineage specific genes in glia and melanocytes [7], [8], [9]. Homozygous loss-of-function mutation in Sox10 is embryonic lethal, but the critical role Sox10 plays in melanocyte development is evident as seen by the reduced number or absence of cells expressing specific markers [10]. Also, heterozygous loss-of-function mutations, including the frame shift mutation in *Dom* mouse, result in melanocytic phenotype with reduced extent of pigmentation in the belly and limb extremities [10], [11], [12]. Given the effect of differentiation program inherent to melanocytes on migration and metastasis, we sought to determine if Sox10 regulates migration and metastatic behaviors of melanoma. Several studies regarding the role of Sox10 in melanoma indicate that Sox10 is expressed in most if not all primary and metastatic melanoma cells and drives the expression of nestin which is correlated with poor prognosis [13], [14], [15]. Interestingly, Agnarsdottir and co-workers showed that inhibition of Sox10 expression with siRNA led to down-regulation of migration in the case of WM115 melanoma cells but not in the case of WM793 cells suggesting that the role of Sox10 is not equivalent in all melanoma cells [16]. This in turn indicates that in order to analyze the role of Sox10 in migration and isolate relevant down-stream target genes, it is necessary to select an appropriate melanoma cell line.

Here, we used RNA interference technique to demonstrate the role of Sox10 on migration in B16F10 melanoma cells and to identify its potential transcriptional regulatory targets. We show that multiple targets of Sox10 including Mc1r, the key signaling factor in skin and hair pigmentation, are involved as effectors in B16F10 melanoma cell migration [17]. We also present data that indicate microphthalmia-associated transcript factor, Mitf, an established target of Sox10 and a critical regulator of melanocyte development, likely mediates much of the effect of Sox10 with regards to migration of B16F10 cells [18], [19], [20]. Finally, using a murine in vivo metastasis model we confirm the involvement of Mc1r in migration and metastasis of melanoma. Together, these data establish a novel group of genes involved in regulation of melanoma migration and metastasis some of which may represent potential novel targets of therapeutic intervention.

## Results

**Figure 1:**
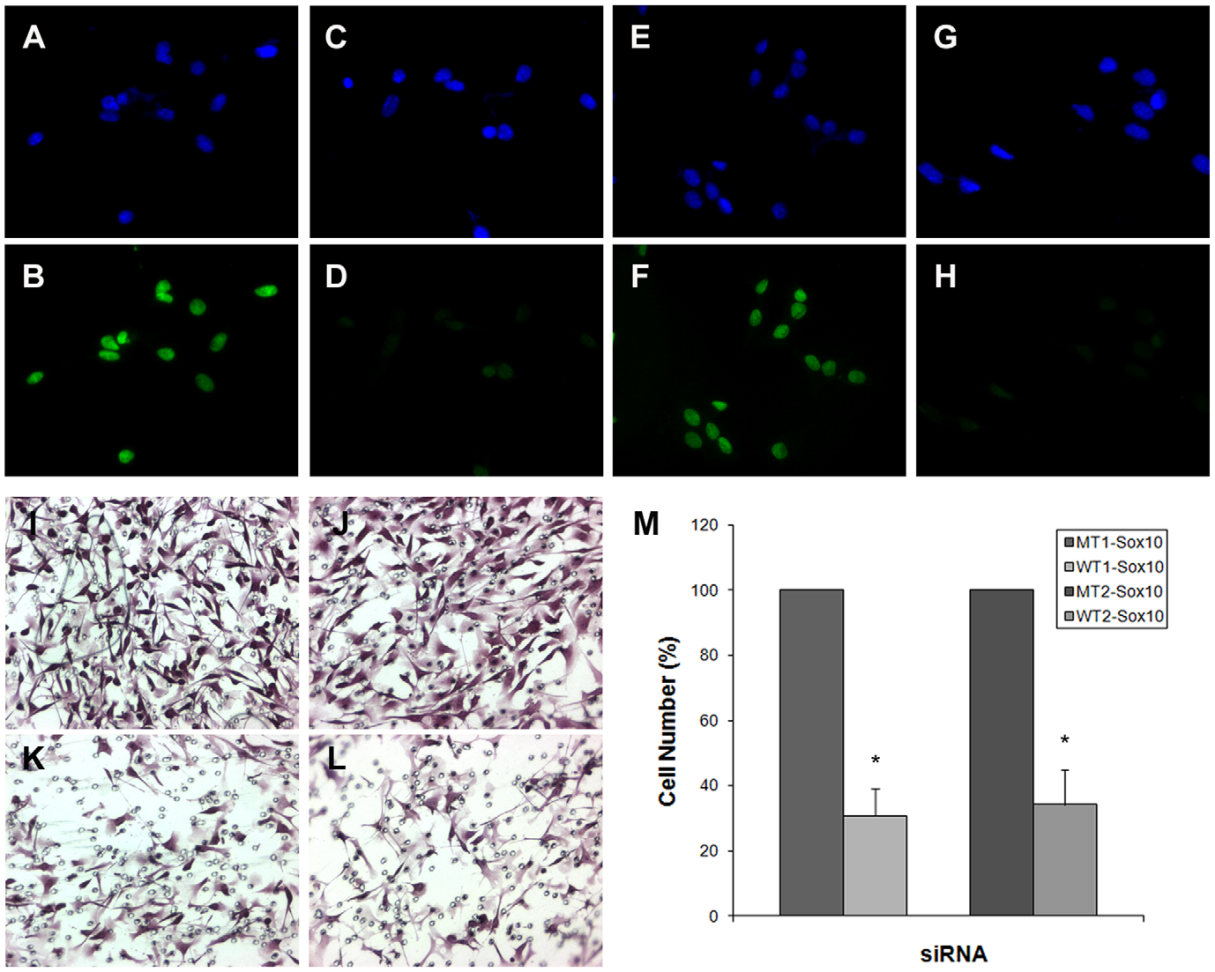
Knockdown of Sox10 leads to inhibition of migration of B16F10 melanoma cells. B16F10 murine melanoma cells were transfected with control siRNAs, MT1-Sox10 (A, B; MT, mutant) and MT2-Sox10 (E, F) or siRNAs specific for Sox10, WT1-Sox10 (C, D; WT, wild type) and WT2-Sox10 (G, H). Nuclei were stained with DAPI (A, C, E, G) and anti-Sox10 antibody (B, D, F, H). Nucleotide sequences of MT1-Sox10 and MT2-Sox10 differ from those of WT1-Sox10 and WT2-Sox10 by 5 nucleotides respectively (see Table S1). Sox10 was down-regulated only with WT siRNAs (D, H) but not with MT siRNAs (B, F). B16F10 cells treated with siRNAs were put to transwell migration assay (I–M). Transfection of WT1-Sox10 (K) and WT2-siRNA (L) led to significant reduction in migration of the cells compared to transfection of MT1-Sox10 (I) or MT2-Sox10 (J). (M) Quantitation of transwell migration assay. The effect of Sox10 knockdown on the number of cells that migrated through the filter pores is shown in percentile relative to the matching control case. Values represent the average of 5 independent trials, and error bars represent standard deviations. The asterisk (*) represents a significant difference with the *p* value of <0.05.

We first sought to determine if Sox10 is involved in regulating migratory behavior of B16F10 melanoma cells. To this end, we transfected B16F10 melanoma cells with two independent siRNAs specific for murine Sox10 (the sequences of siRNAs are listed in Table S1). In 24 hours, Sox10 was undetectable in most of the cells while the expression was maintained in the cells transfected with control siRNAs (Fig. 1A–H). Importantly, cells transfected with the Sox10-specific siRNAs showed a significant reduction in migration compared to the control cells in transwell migration assays (Fig. 1I–M). This was not due to the varying replating efficiency as we observed no difference between the cell populations (data not shown). We tested two pairs of control and specific siRNAs for Sox10 to rule out the ‘off-target’ effects. The two pairs showed essentially the same effects on Sox10 expression and on cell migration. We examined if down-regulation of Sox10 induced apoptosis or senescence which could explain the observed difference between the cell populations. TUNEL assay and senescence-associated β-galactosidase assay were carried out and showed that neither of the two processes was taking place (Figs. S1 and S2).

**Figure 2:**
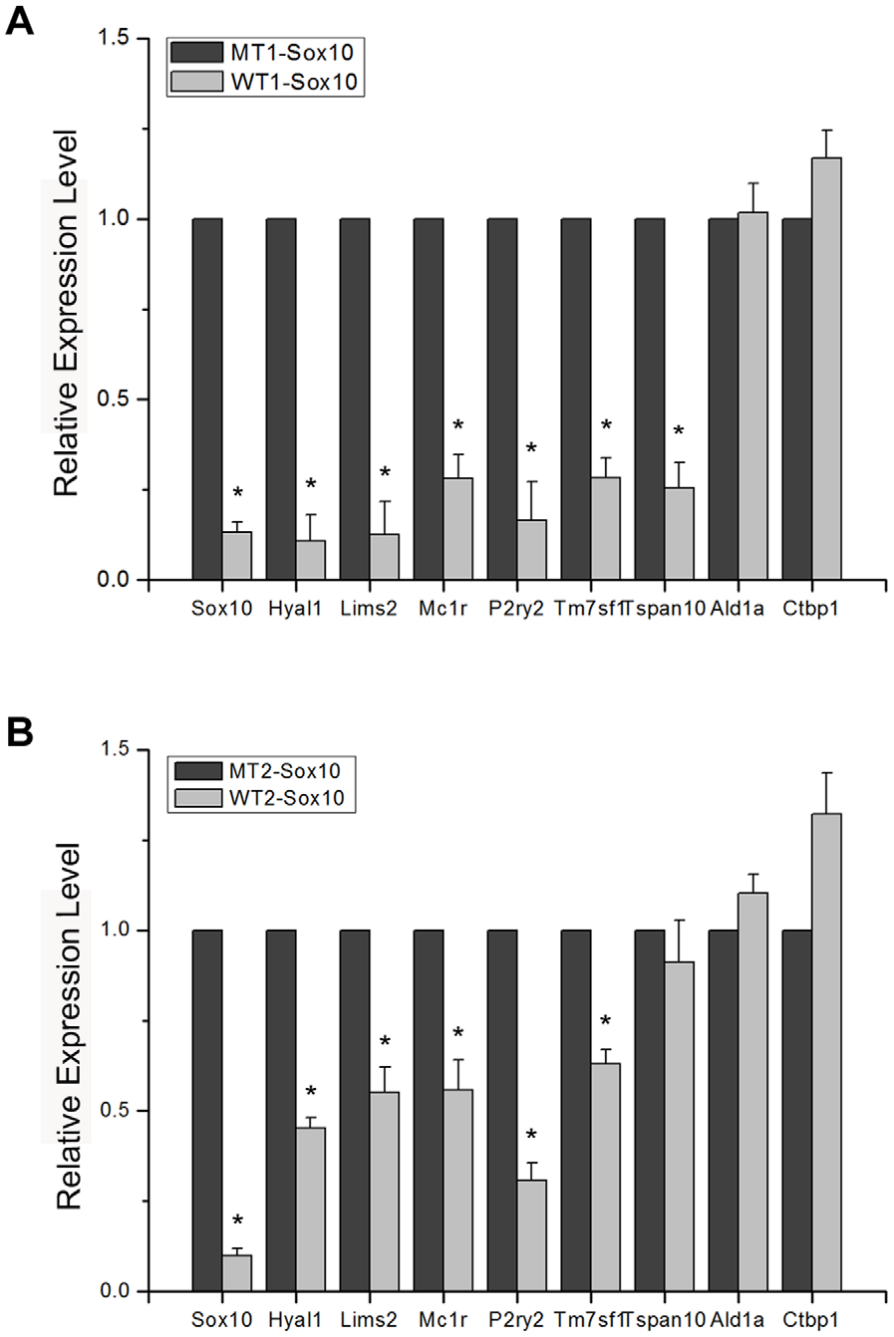
Confirmation of microarray expression profiling. (A) Quantitative real time RTPCR assays were carried out using B16F10 cells transfected with the MT1-Sox10 or WT1-Sox10. A subset of genes that showed down-regulation by WT1-Sox10 in the microarray assay by 2.5 fold or higher in all triplicates (Table 1) and two non-target genes whose expression levels were unchanged (Ald1a and Ctbp1) were used to validate the results from the microarray assay. The effect of Sox10 knockdown by the specific siRNA on the expression level of each target gene is expressed relative to that of the control siRNA after normalization with GAPDH expression level. Values represent the average of three independent real-time PCR experiments each carried out in duplicates, and error bars represent standard deviations. (B) Real time RTPCR carried out with MT2-Sox10 and WT2-Sox10. The asterisk (*) represents a significant difference with the *p* value of <0.05.

Sox10, a transcription factor, presumably regulates the expression of other genes that function as the “effector” genes of migration of melanoma cells. We thus performed a microarray screen using siRNA-treated cells in order to identify candidate Sox10-regulated genes involved in melanoma cell migration (Table 1). The complete microarray data from triplicate trials are deposited in the Gene Express Omnibus (GEO) database [GEO: GSE25501]. Table 1 shows genes down-regulated by more than 2.5 fold in all triplicates upon transfection of a Sox10-specific siRNA. Sox10 itself showed approximately three fold down-regulation as the result of specific siRNA transfection. One of the established targets of Sox10 in melanocytes, Mitf was also shown to be down-regulated by about three fold attesting to the validity of results from the screen [18], [19]. We confirmed the results from the microarray screen using quantitative RTPCR for several down-regulated and unaffected genes using both pairs of control and Sox10-specific siRNAs (Fig. 2; sequences of oligonucleotide primers are listed in Table S2).

**Table 1:** List of genes down-regulated by WT1-Sox10.

Accession No.	Gene name	Symbol	Fold change
BC158603	ATP-binding cassette, sub-family A (ABC1), member 12	Abca12	−6.04
NM_009308	synaptotagmin IV	Syt4	−4.74
NM_175475	cytochrome P450, family 26, subfamily b, polypeptide 1	Cyp26b1	−4.08
AF154337	transmembrane 7 superfamily member 1	Tm7sf1	−4.03
NM_000086	proteolipid protein	Plp	−4.00
NM_009280	synovial sarcoma translocation, Chromosome 18	Ss18	−3.73
NM_144862	LIM and senescent cell antigen like domains 2	Lims2	−3.58
NM_145363	oculospanin	Ocsp	−3.57
NM_177740	RGM domain family, member A	Rgma	−3.23
NM_172584	inositol 1,3,4-triphosphate 5/6 kinase	Itpk1	−3.19
NM_009177	sialyltransferase 4A (beta-galactoside alpha-2,3-sialytransferase)	Siat4a	−3.15
NM_008618	malate dehydrogenase 1, NAD (soluble)	Mdh1	−3.14
NM_008773	purinergic receptor P2Y, G-protein coupled 2	P2ry2	−3.13
NM_008601	microphthalmia-associated transcription factor	Mitf	−3.10
NM_145933	beta galactoside alpha 2,6 sialyltransferase 1	St6gal1	−3.05
NM_144862	LIM and senescent cell antigen like domains 2	Lims2	−3.04
NM_008125	gap junction membrane channel protein beta 2	Gjb2	−2.99
NM_001014973	sorting nexin 13	Snx13	−2.99
XM_001471531	mutated in colorectal cancers	Mcc	−2.93
NM_001033217	prickle like 1 (Drosophila)	Prickle1	−2.92
NM_011437	SRY-box containing gene 10	Sox10	−2.90
NM_008889	protein phosphatase 1, regulatory (inhibitor) subunit 14B	Ppp1r14b	−2.89
NM_008963	prostaglandin D2 synthase (brain)	Ptgds	−2.85
NM_008317	hyaluronidase 1	Hyal1	−2.85
NM_010777	myelin basic protein	Mbp	−2.82
NM_028238	Rab38, member of RAS oncogene family	Rab38	−2.81
NM_011160	protein kinase, cGMP-dependent, type I	Prkg1	−2.79
NM_011594	tissue inhibitor of metalloproteinase 2	Timp2	−2.79
NM_021883	tropomodulin 1	Tmod1	−2.76
NM_053077	membrane associated transporter protein	Matp	−2.76
NM_026794	differentially expressed in B16F10 1	Deb1	−2.76
NM_028017	N-ethylmaleimide sensitive fusion protein attachment protein gamma	Napg	−2.73
NM_173401	F-box protein 44	Fbxo44	−2.72
NM_008156	glycosylphosphatidylinositol specific phospholipase D1	Gpld1	−2.71
NM_010271	glycerol-3-phosphate dehydrogenase 1 (soluble)	Gpd1	−2.70
NM_007943	epidermal growth factor receptor pathway substrate 15	Eps15	−2.68
NM_011797	carbonic anhydrase 14	Car14	−2.65
AF035643	vesicle-associated membrane protein 5	Vamp5	−2.63
NM_013807	polo-like kinase 3 (Drosophila)	Plk3	−2.62
NM_008559	melanocortin 1 receptor	Mc1r	−2.60
NM_007502	ATPase, Na+/K+ transporting, beta 3 polypeptide	Atp1b3	−2.59
NM_008139	guanine nucleotide binding protein, alpha q polypeptide	Gnaq	−2.59
NM_008397	integrin alpha 6	Itga6	−2.58
NM_011923	angiopoietin-like 2	Angptl2	−2.57
NM_145439	transmembrane channel-like gene family 6	Tmc6	−2.56
NM_053110	glycoprotein (transmembrane) nmb	Gpnmb	−2.56
NM_021889	synaptotagmin 9	Syt9	−2.56
NM_007905	polyhomeotic-like 1 (Drosophila)	Phc1	−2.56
NM_080793	SET domain-containing protein 7	Set7	−2.55
NM_172992	putative homeodomain transcription factor 2	Phtf2	−2.54
NM_176996	smoothened homolog (Drosophila)	Smo	−2.54
NM_011390	solute carrier family 12, member 7	Slc12a7	−2.52
NM_130886	caspase recruitment domain family, member 14	Card14	−2.52
NM_175445	Ras association (RalGDS/AF-6) domain family 2	Rassf2	−2.50
NM_010164	eyes absent 1 homolog (Drosophila)	Eya1	−2.50

Fold change is in comparison to MT1-Sox10 transfected cells. Microarray screening was carried out in triplicates. Genes that show down-regulation by 2.5 fold or higher in all triplicates are listed. Oculospanin (oscp) is a synonym for tetraspanin 10 (Tspan10).

**Figure 3:**
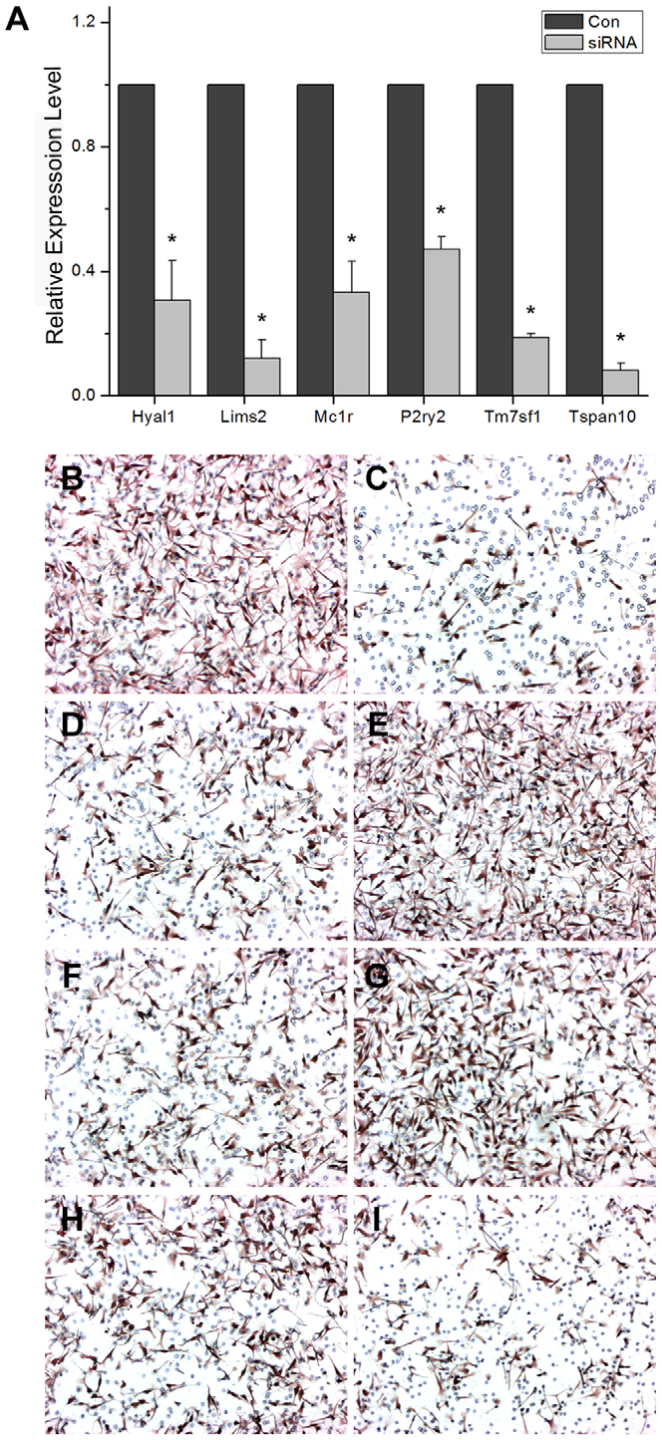
Targets of Sox10 regulate migration of B16F10 melanoma cells. (A) Quantitative RTPCR assay was carried out to test the efficacy of siRNAs directed for the selected genes. For each gene, the knockdown effect of the specific siRNA is expressed relative to that of a universal control siRNA (siCONTROL Non-Targeting siRNA #2) after normalization with GAPDH expression level. Values represent the average of three independent real-time PCR experiments each carried out in duplicates, and error bars represent standard deviations. The asterisk (*) represents a significant difference with the *p* value of <0.05. (B-I) B16F10 cells were treated with the universal control siRNA, WT1-Sox10 or a specific siRNA for each of the selected Sox10 target genes and subjected to transwell migration assay: Control (B), Sox10 (C), Hyal 1 (D), Lims2 (E), Mc1r (F), P2ry2 (G), Tm7sf1 (H), and Tspan10 (I). Representative results are presented.

In identifying effector genes of migration that are downstream to Sox10, we focused on the subset of genes whose protein products are either localized to cellular membrane or associated with cell motility as genes of such categories are likely to be directly involved in cellular migration. To this end, 6 genes, Tm7sf1, Lims2, Tspan10, P2ry2, Hyal1, and Mc1r were selected. While some of these genes have previously been implicated in cell migration and/or metastasis, others have not been functionally characterized [21], [22], [23]. We generated a specific siRNA for each of these genes and tested for their efficacy in reducing the target mRNA and in inhibiting migration of B16F10 cells. Quantitative RTPCR analysis showed that all of the siRNAs were effective in down-regulating their target genes (Fig. 3A). Transwell migration assays showed that down-regulating Hyal1, Tspan10, and Mc1r resulted in clearly reduced migration of the melanoma cells (Fig. 3D, 3F, 3I). In contrast, no visible effects were seen with siRNAs for Lims2, P2ry2 and Tm7sf1 (Fig. 3E, 3G, 3H). A universal control (Fig. 3B) and WT1-Sox10 (Fig. 3C) were used as negative and positive controls respectively. We thus define multiple potential effectors of migration downstream to Sox10 in melanoma cells.

**Figure 4:**
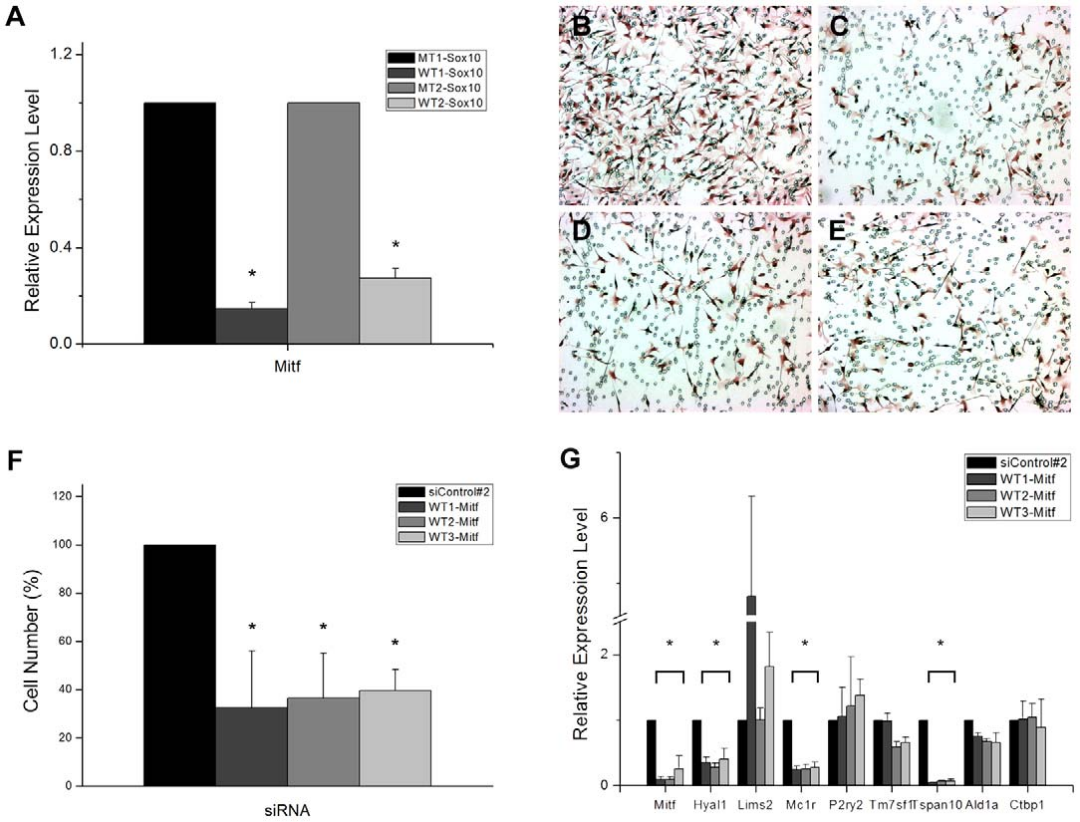
Mitf mediates the effect of Sox10 on cell migration. (A) Quantitative real time RTPCR assays were carried out using B16F10 cells transfected with the mutant or wild type Sox10 siRNAs. Mitf expression level was determined to confirm the microarray data. The effect of Sox10 knockdown by each of the two specific siRNAs on the expression level of Mitf is expressed relative to that of the corresponding control siRNA after normalization with GAPDH expression level. Values represent the average of three independent real-time PCR experiments each carried out in duplicates, and error bars represent standard deviations. (B–F) B16F10 cells were treated with the universal control siRNA (B) or one of the specific siRNAs for Mitf, WT1-Mitf (C), WT2-Mitf (D), and WT3-Mitf (E) and subjected to transwell migration assay. (F) Quantitation of transwell migration assay. The effect of Mitf knockdown on the number of cells that migrated through the filter pores is shown in percentile relative to the control case. Values represent the average of 5 independent trials, and error bars represent standard deviations. (G) Quantitative real time RTPCR assays were carried out using B16F10 murine melanoma cells transfected with the control siRNA or with one of the three Mitf-specific siRNAs. Effects on the expression level of Mitf, selected Sox10-target genes, and two non-target genes (Ald1a and Ctbp1) were examined. The effect of Mitf knockdown by the specific siRNA is expressed relative to that of the control siRNA after normalization with GAPDH expression level. Values represent the average of three independent real-time PCR experiments each carried out in duplicates, and error bars represent standard deviations. The asterisk (*) represents a significant difference with the *p* value of <0.05.

Much of the role Sox10 plays in melanocytes is supposedly mediated by one of its established direct targets, Mitf, a transcription factor belonging to the basic-helix-loop helix leucine zipper transcription factor family [24]. As described above, Mitf is down-regulated upon siRNA-mediated down-regulation of Sox10 which was confirmed by RTPCR analysis as well (Fig. 4A). We sought to determine if Mitf is also involved in regulating migration of melanoma cells. A battery of specific siRNAs was designed and tested for efficacy in down-regulating Mitf by RTPCR (Fig. 4G) and in inhibiting migration of melanoma cells (Fig. 4B–F). Indeed, a significant reduction in migration was seen when the specific siRNAs were transfected. Apoptosis and senescence were not responsible for the apparent differences among the cell populations (Figs. S1 and S2). To test if the 6 target genes of Sox10 were regulated by Mitf, their expression levels were examined in the cells transfected with the Mitf siRNAs. Consistent with a previous report, Mc1r was regulated by Mitf [25]. Of the remaining 5 genes, Hyal1 and Tspan10 were shown to be clearly downstream to Mitf while Lims2, P2ry2, and Tm7sf1 did not appear to be so (Fig. 4G). These data thus propose a novel regulatory network initiated by Sox10 and mediated by Mitf that culminates in a group of effector genes for migration of melanoma.

We examined two other mouse melanoma cell lines, Cloudman S91 and Melan-a for the involvement of Sox10 in migration (Fig. S4). Although various transfection protocols were attempted, the efficiency of Sox10 down-regulation by specific siRNA was significantly less in these cells than in B16F10 (Fig. 2A), and consequent down-regulation of Mitf and Mc1r was also clearly less efficient. Interestingly, migration was not at all affected by down-regulation of Sox10 in both cell lines (see Discussion).

**Figure 5:**
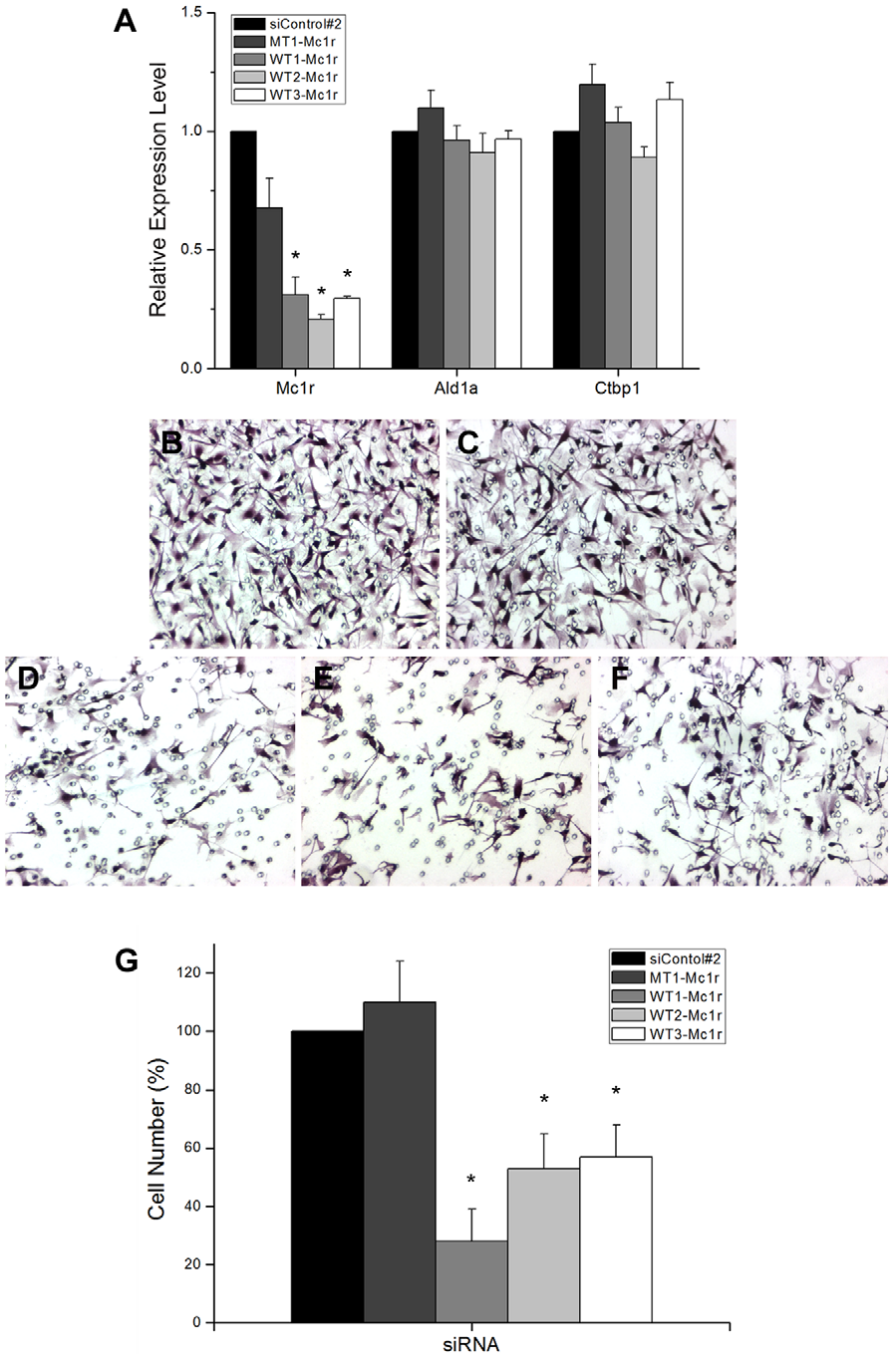
Mc1r promotes migration of melanoma cells. (A) Quantitative Real time RTPCR assays were carried out using B16F10 murine melanoma cells transfected with the universal control siRNA, MT1-Mc1r, or one of the three siRNAs specific for Mc1r, WT1-Mc1r, WT2-Mc1r, and WT3-Mc1r. The nucleotide sequence of MT1-Mc1r differs from that of WT1-Mc1r by 5 nucleotides. The expression levels of Mc1r, Ald1a, and Ctbp1 were examined. The effect of Mc1r knockdown is expressed relative to that of the universal control siRNA after normalization with GAPDH expression level. Values represent the average of three independent real-time PCR experiments each carried out in duplicates, and error bars represent standard deviations. (B–G) B16F10 cells were treated with the universal control siRNA (B), MT1-Mc1r (C), WT1-Mc1r (D), WT2-Mc1r (E), or WT3-Mc1r (F) and put to transwell migration assay. (G) Quantitation of transwell migration assay. The effect of Mc1r knockdown on the number of cells that migrated through the filter pores is shown in percentile relative to the universal control case. Values represent the average of 5 independent trials, and error bars represent standard deviations. The asterisk (*) represents a significant difference with the *p* value of <0.05.

Subsequent in-depth analyses were focused on Mc1r. One well-established function of Mc1r in melanocytes and melanoma is to mediate the melanogenic signal [17]. Interestingly, several studies have reported that melanocortin stimulating hormone (MSH), a melanogenic ligand of Mc1r, inhibits migration and metastasis of melanoma cells [26], [27], [28]. This was confirmed using transwell migration assay in our hands as well (data not shown). In contrast, a more recent study reported that another ligand of Mc1r, agouti signal protein stimulated migratory activity of melanoma cells [29]. Collectively, these data indicate that Mc1r is somehow involved in mediating migratory signaling in melanoma. We thus generated additional Mc1r-specific siRNA molecules for transfection into B16F10 melanoma cells. Quantitative RTPCR showed that all three specific siRNAs reduced the level of Mc1r transcript (Fig. 5A), and transwell migration assay showed that each of the siRNAs led to reduction in melanoma migration (Fig. 5B–G). Once again, Apoptosis and senescence were not responsible for the apparent differences among the cell populations (Figs. S1 and S2).

**Figure 6:**
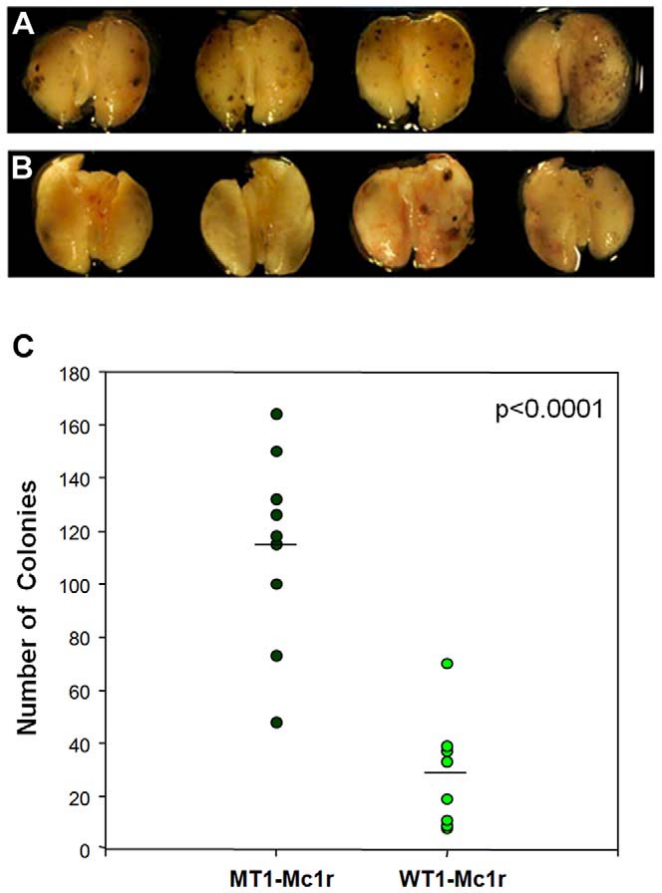
Confirmation of the role of Mc1r on cell migration using an in vivo metastasis model. (A, B) Effect of Mc1r knockdown on the development of pulmonary metastatic colony was determined. B16F10 melanoma cells were treated with MT1-Mc1r (A) or WT1-Mc1r (B) and injected into tail vein of C57BL/6 mice. Representative lungs harvested after 18 days are shown. (C) B16F10 colonies visible on the lung surface were counted and plotted. N = 9 for MT1-Mc1r and N = 8 for WT1-Mc1r. The significance of difference (p<0.0001) was determined by *t*-test.

We next examined the potential role of Mc1r in metastasis of melanoma using an established in vivo model. Injection of B16F10 melanoma cells into tail veins of congenic C57BL/6 mice leads to pulmonary metastasis of these cells which can be quantitated by counting clonal colonies. Involvement of specific genes in the metastasis of B16 melanoma cells has been tested using transient transfection of siRNAs in this system [30], [31]. From the siRNAs tested in vitro, we selected WT1-Mc1r which was the highly effective in down-regulating Mc1r expression and melanoma migration. As for the control, MT1-Mc1r, whose sequence contains 5 mismatches to WT1-Mc1r, was used. Treatment of B16F10 cells with these siRNAs did not differentially affect the cell growth as evidenced by cell cycle phase analysis and MTT assay (data not shown). We also examined cellular proliferation by BrdU labeling at various time points subsequent to siRNA transfection upto 18 days and showed that at all time points the two cell populations were equivalent in this regard (Fig. S3). Cells, transfected either with MT1-Mc1r or WT1-Mc1r 24 hours before injection, were injected into tail veins of multiple recipient mice, and lungs were harvested and examined visually for melanoma clones after 18 days. There was a clear and significant difference in the number of clones between the two populations of mice (Fig. 6A–C). Taken together with the data from in vitro experiments, these results support the hypothesis that Mc1r is required for efficient migration and thereby the metastasis of melanoma cells.

## Discussion

The notion that key genes in development and differentiation of melanocytes are also involved in metastasis of melanoma strongly suggests that elaboration of molecular function of such genes would reveal much about the mechanism of metastasis [4]. In this study, we show that the cascade of gene expression initiated by Sox10 and subsequently mediated in part by Mitf likely represents an important regulatory axis of migration and metastasis in a subset of melanoma cases. These two transcription factors have been established as key regulators of differentiation, proliferation, and survival of melanocytes. Such involvement in multiple aspects has in turn made it difficult to identify and analyze their role in migration of developing melanocytes. In this regard, B16F10 melanoma cells, highly migratory and impervious to down-regulation of Sox10 and Mitf for survival at least short term, have been useful. Strictly speaking, the current study does not warrant the extension of the novel role of Sox10 and Mitf to developing melanocytes. However, at least in the case of zebrafish it has been reported that mutations in Sox10 inhibit migration of melanoblasts prior to their death by apoptosis [32]. Plus, it would be an extraordinary coincidence if melanoma co-opted the two lineage specific transcription factors for functions not existent in the precursor cell type.

It has been questioned how these two genes, especially Mitf, could be involved in such multiple aspects of melanocyte biology some of which appear to be mutually in conflict [20]. For example, how they impart positive effects on both proliferation and differentiation of melanocytes has yet to be answered although the dosage effect has been proposed as a partial explanation. Our study adds on to the complexity by implicating these two transcription factors in migration of melanocytes and melanoma. One possible approach in dissecting the situation is to find targets of Sox10 and Mitf that are involved uniquely in migration but not in survival, proliferation, or differentiation. The genes identified in this study whose expression is altered by suppression of Sox10 should comprise the pool of genes for such future analyses. Clearly, genes that are up-regulated also represent potential regulatory effectors of migration although they are not examined in this study.

It should be noted that whether the Sox10-Mitf axis is functional in regulating migratory behavior of all melanoma is questionable at this stage. First, Sox10 appears to be broadly expressed in both metastatic and non-metastatic melanomas [13], [14]. Secondly, in a recent study, Agnarsdottir and co-workers showed that down-regulation of Sox10 differentially affects migration in different melanoma cell lines [16]. Consistently, our preliminary data suggest that Sox10 is not involved in promoting migration in Cloudman S91 and Melan-a cells unlike in B16F10 cells (Fig. S4). We cannot rule out that this lack of regulatory effect results from the less efficient down-regulation of Sox10 which we were able to achieve in these cells. Still, that no effect was seen at all in migration strongly suggests that an alternative explanation should be sought. Sox10-Mitf axis may be co-opted for different roles in different melanoma cells, and it may thus be possible or even necessary to group melanomas into distinct sets based on the role of Sox10-Mitf axis. Another issue to be addressed further concerns the exact nature of migration regulated by Sox10-Mitf axis. Our preliminary data indicates that both chemotactic and chemokinetic migrations are promoted in B16F10 cells by Sox10 as migration was down-regulated by siRNA treatment even when identical culture media were used in upper and lower chambers in transwell assays (Fig. S5). However, the degree of down-regulation was less than that with the use of differential media suggesting chemotactic component is also present.

It is interesting that the three of the six Sox10 target genes studied here which Mitf also appears to regulate are those that affect migration. This seems to indicate that much of the effect of Sox10 on melanoma migration is in fact mediated by Mitf although an exhaustive study needs to be carried out with more genes from the microarray screen. Hyal1, a hyaluronidase, cleaves hyaluronan which is a polysaccharide composed of repeating disaccharides of glucouronic acid and N-acetylglucosamine [33]. Although best known as a component of extracellular matrix, short hyaluronan fragments generated from cleavage by hyaluronidase have been shown to participate in cell signaling pathways including those that regulate proliferation and migration [33], [34]. In at least one report, hyaluronan fragments have been shown to induce cytokine and metalloprotease expression and promote motility of melanoma [35]. It would be interesting to test in our system if Hyal1, regulated by Sox10-Mitf axis, is in fact cleaving hyaluronan to activate a signaling pathway in promotion of cell migration. Tspan10 belongs to a family of membrane proteins known as tetraspanins [36]. Little is known about the function of Tspan10 although the existence of orthologues throughout vertebrates strongly suggests that it has a conserved and important function. The family members are variously implicated in regulation of cell signaling, adhesion, motility, fusion, and viral infection [36]. Thus, it would not be surprising if Tspan10 partakes in regulating migration of melanoma as shown in our study although detailed mechanisms need further analyses.

We propose Mc1r as an important mediator gene of migratory signaling in this study. Aside from the in vitro assays for its effect on migration, we also performed in vivo assay and showed that Mc1r can potentially regulate metastatic behavior of melanoma cells. It should be noted that although a popular model, the experimental system based on intravenous delivery of cells represents examination of extravasation rather than overall metastasis [30], [31]. A more rigorous model will be needed to confirm that Mc1r can affect exit from the primary tumor, the first step of metastasis.

Interestingly, it appears that Mc1r can bind multiple ligands and can impart at least two distinct signals, one melanogenic and another pro-migratory. First, agouti signal protein, a well-established ligand of Mc1r has been shown to stimulate migration of melanoma cells to a significant level [29]. Second, α-MSH, another well-established ligand of Mc1r inhibits migration and metastasis of melanoma cells [26], [27], [28]. Also, a member of the β-defensin family, previously studied mostly for its role in immunity, has been recently reported to be a specific ligand of Mc1r, [37]. Consistently, it has been reported that Mc1r is coupled to more than one signaling pathways and that Mc1r shows an agonist-independent constitutive activity [17], [38]. It is then possible that constitutive signaling initiated by Mc1r in the absence of melanogenic ligand is promigratory rather than melanogenic although it is very difficult if not impossible to show that our in vitro system is entirely devoid of any known or unknown Mc1r ligands. It goes without saying that it would be important down the road to dissect the pro-migratory signaling pathway of Mc1r apart from the melanogenic signaling pathway. Components of pro-migratory pathway represent potential targets for inhibition of metastasis which apparently is the key to surviving the scourge of melanoma.

## Materials and Methods

### Cell culture

Mouse melanoma cell line B16F10 cells were obtained from ATCC and cultured in DMEM (WelGENE Inc.) supplemented with 10% fetal bovine serum (FBS; Hyclone), 1% penicillin and 1% streptomycin in a humidified chamber with 5% CO_2_ at 37°C. HT1080 cells were grown in MEM (Invitrogen) supplemented with 10% fetal bovine serum, 1 mM sodium pyruvate and 1% penicillin and 1% streptomycin in a humidified chamber with 5% CO_2_ at 37°C.

### Immunostaining

Cells were fixed in 4% paraformaldehyde in PBS. Immunocytochemistry with the monoclonal anti-Sox10 antibody has been described [39]. For DAPI staining, cells were incubated with DAPI (Sigma) at 0.1 µg/ml in PBS at RT for 10 min. Cells were viewed and photographed using Axiovert 200 epiflourescence microscope (Carl Zeiss Inc.) equipped with a digital imaging system.

### siRNA experiments

Synthetic 21-nucleotide RNA duplexes were purchased from Dharmacon Research Inc. Target sequences for specific genes were selected using siDESIGN® Center tool (http://www.dharmacon.com/designcenter/designcenterpage.aspx). As a universal control, siCONTROL Non-Targeting siRNA#2 was used. Other control mutant (MT) siRNAs were designed to contain 5 nucleotide mismatches to corresponding wild type (WT) siRNAs. Cells were trypsinized and replated 1 day prior to transfection at the density of 5×10^5^ cells per 60 mm dish. The siRNAs with the final concentration of 200 nM were transfected into B16F10 cells using Oligofectamine (Invitrogen) according to the manufacturer's instruction. siRNAs were typically applied for 24 hours.

### Gene expression profiling

Total RNA was prepared using an RNeasy Mini Kit (QIAGEN) according to the manufacturer's instructions and subsequently processed to yield biotinylated cRNA using the Ambion Illumina RNA amplification kit (Ambion, Austin, USA) according to the manufacturer's instructions. The labeled cRNA preparations were applied to Mouse-6 Expression BeadChip (Illumina, Inc). Detection of array signal was carried out using Amersham fluorolink streptavidin-Cy3 (GE Healthcare Bio-Sciences, Little Chalfont, UK) following the bead array manual. Arrays were scanned with an Illumina Bead Array Reader confocal scanner according to the manufacturer's instructions. Raw data were extracted using the software provided by the manufacturer, Illumina BeadStudio (Gene Expression Module). Array data were filtered by detection value > = 0.66 (similar to signal to noise) in at least 80% samples (we applied a filtering criterion for data analysis; higher signal value was required to obtain a detection p-value<0.05). Selected gene signal value was transformed by logarithm and normalized by quantile method. The comparative analyses between test samples and control samples were carried out using fold-change. The complete microarray data from triplicate trials were deposited in the Gene Express Omnibus (GEO) database [GEO: GSE25501]. All data are MIAME compliant. Go-ontology analysis for significant probe list was performed using PANTHER (http://www.pantherdb.org).

### Real time RTPCR

Total RNA was extracted using an RNeasy Mini Kit (QIAGEN) according to the manufacturer's instructions. cDNA synthesis was carried out with SuperScript® First-Strand Synthesis System for RT-PCR (Invitrogen) using oligo-dT primers following the manufacturer's instructions. Quantitative analyses of gene expression level were performed by real-time PCR with SYBR Green Master mix (Applied Biosystems). PCR was carried out on an ABI Prism 7300 Sequence Detection System using a two-step thermal cycling protocol of 40 cycles of 95°C for 15 s and 60°C for 1 min, preceded by an initial 95°C for 10 min for activation of AmpliTaq Gold® DNA polymerase. The expression value of tested genes was calculated using the delta Ct method with normalizing to the GAPDH expression level. Primer pairs used for PCR are listed in Table S2.

### Transwell migration assay

Transwell migration assays were performed using transwell chambers (8 µm polycarbonate membrane, Corning). Lower-chamber side filter was coated for 1 hour with 0.1% gelatin B. After 24 hours of siRNA treatment, B16F10 cells were harvested in DMEM containing 2% FBS. Typically, 1×10^5^ cells were seeded in the upper chamber which was placed over the lower chamber containing HT1080-conditioned media. Migration was allowed to proceed for 6 hours at 37°C, and cells were fixed with 70% Methanol and stained with 0.5% EosinB-Hematoxylin solution. Cells remaining on the upper-chamber side of the filter were removed with cotton swabs. The number of migrated cells was determined by counting stained cells from multiple randomly selected microscopic visual fields.

### In vivo metastasis assay

B16F10 cells, treated with WT1-Mc1r or MT1-Mc1r siRNA for 24 hours were washed twice with PBS and detached with trypsin. After serum inactivation, cells were again washed and resuspended in PBS. Male C57BL/6 mice, 8 weeks old, were injected in the tail vein with 2×10^6^ cells in 400 µl. After 18 days, mice were euthanized by standard carbon dioxide asphyxiation. The lungs were removed and rinsed, and the pulmonary metastatic colonies of B16F10 cells were counted by visual inspection. All animal studies were conducted in accordance with the IACUC guidelines and were approved by the IACUC committee at Ewha Womans University (approval ID: ELAGC-09-1019 and IACUC 2010-15-2).

More information is available in Methods S1.

## Supporting Information

Figure S1 **TUNEL assay.** B16F10 cells were transfected with indicated siRNAs for 24 hours and replated as in the transwell migration assay. TUNEL assay was performed using TUNEL-Enzyme and TUNEL-Label (Roche). DNase I treated cells were used as positive controls for TUNEL staining. Cells were also stained with specific antibodies to confirm down-regulation of Sox10 (A) and MITF (B). For Mc1r (C), RTPCR was performed (D) in duplicates to confirm the down-regulation (see also Fig. 4G and Fig. 5A). Ald1a and Crbp1 are negative controls for siRNA treatment for Mc1r.
(PDF)

Figure S2 **Cell staining for senescence-associated beta-galactosidase activity.** B16F10 cells were transfected with indicated siRNAs for 24 hours and replated as in the transwell migration assay. For the positive control, B16F10 cells treated with 50 µM doxorubicin for 5 days were used. Typical X-gal staining and morphological change during senescence are seen only in doxorubicin treated cells.
(PDF)

Figure S3 **BrdU labeling assay.** B16F10 cells were transfected with MT1-Mc1r or WT1-Mc1r and labeled with BrdU for 4 hours at indicated days post transfection. Cells were immunostained for BrdU (A), and the percentages of positive nuclei were determined after DAPI staining. No difference between the two cell populations was observed. (B) Values represent the average of two independent trials, and error bars indicate standard deviation (SD).
(PDF)

Figure S4 **Effect of Sox10 down-regulation on migration of Cloudman S91 and Melan-a melanoma cells.** (A) Quantitative real time RTPCR assays were carried out using Cloudman S91 cells transfected with the MT1 or WT1 Sox10 siRNA. Values represent the average of three independent real-time PCR experiments each carried out in duplicates, and error bars represent standard deviations. The asterisk (*) represents a significant difference with the *p* value of <0.05. Cloudman S91 cells were treated with the MT1 (B) or WT1 (C) Sox10 siRNA and subjected to transwell migration assay. (D) The Graph represents quantitation of transwell migration assay. The effect of Sox10 knockdown on the number of cells that migrated through the filter pores is shown in percentile relative to the control case. Values represent the average of 4 independent trials, and error bars represent standard deviations. (E) Quantitative real time RTPCR assays were carried out using Melan-a cells transfected with the MT1 or WT1 Sox10 siRNA. Values represent the average of three independent real-time PCR experiments each carried out in duplicates, and error bars represent standard deviations. The asterisk (*) represents a significant difference with the *p* value of <0.05. Melan-a cells were treated with the MT1 (F) or WT1 (G) Sox10 siRNA and subjected to transwell migration assay. (H) The Graph represents quantitation of transwell migration assay. The effect of Sox10 knockdown on the number of cells that migrated through the filter pores is shown in percentile relative to the control case. Values represent the average of 4 independent trials, and error bars represent standard deviations.
(PDF)

Figure S5 **Chemokinetic transwell migration assay.** B16F10 cells were transfected with MT1-Sox10 (A) or WT1-Sox10 (B) and subjected to transwell assays with identical media in upper and lower chambers to assess chemokinetic migration. Migration is inhibited in WT1-Sox10 transfected cells. (C) Values represent the average of 5 independent trials, and error bars represent standard deviations.
(PDF)

Methods S1 **Supplementary materials and methods. More information on the TUNEL Assay, Senescence-associated beta-galactosidase activity assay, and BrdU labeling.**
(DOC)

Table S1 **List and target sequence of siRNAs used for RNA interference assay.**
(DOC)

Table S2 **List and sequence of oligonucleotide primers used for real time RTPCR.**
(DOC)
