# Supplementary figures and images for: Sox10 Controls Migration of B16F10 Melanoma Cells through Multiple Regulatory Target Genes
Source: PLoS One. 2012 Feb 21;7(2):e31477. doi: 10.1371/journal.pone.0031477 (PMC3283624; doi:10.1371/journal.pone.0031477)

A

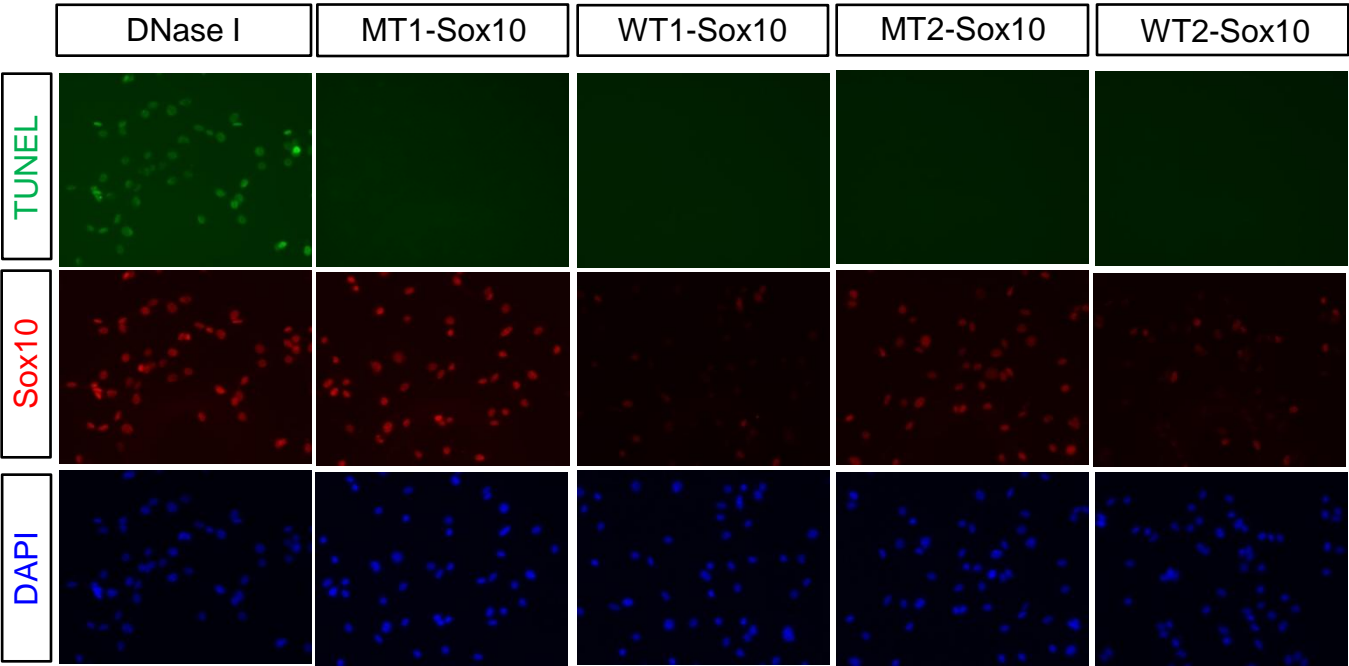

B

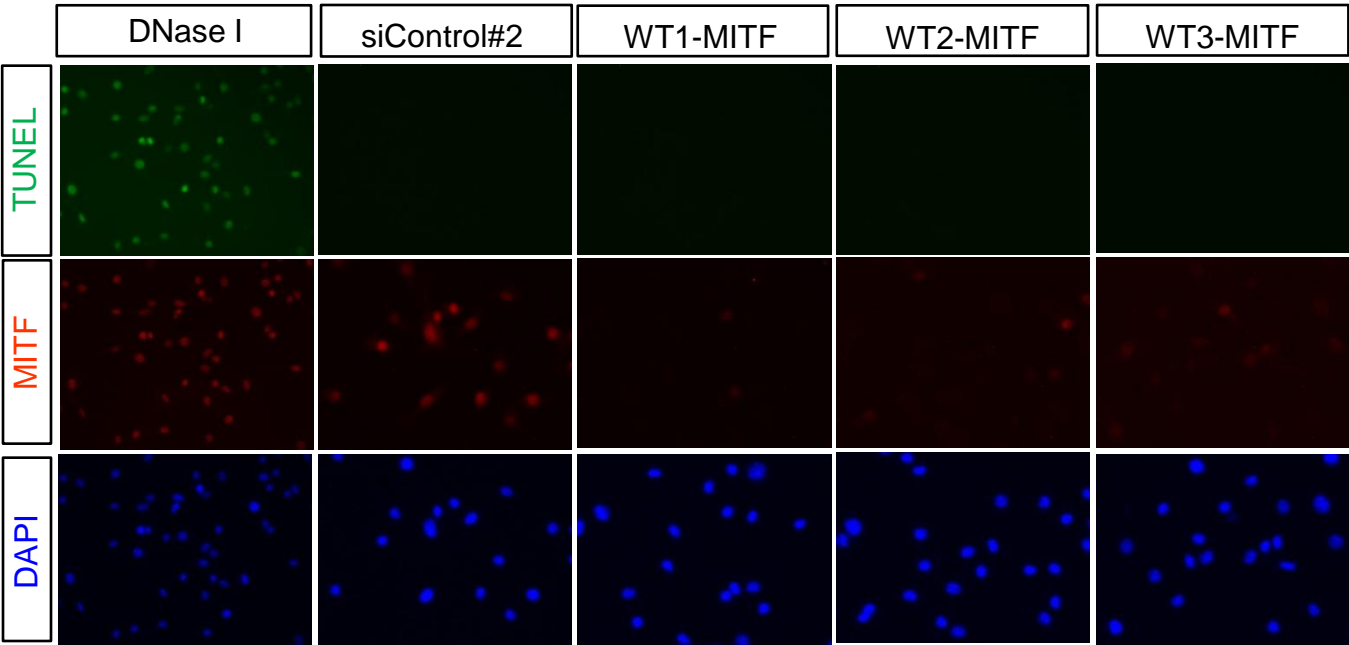

C

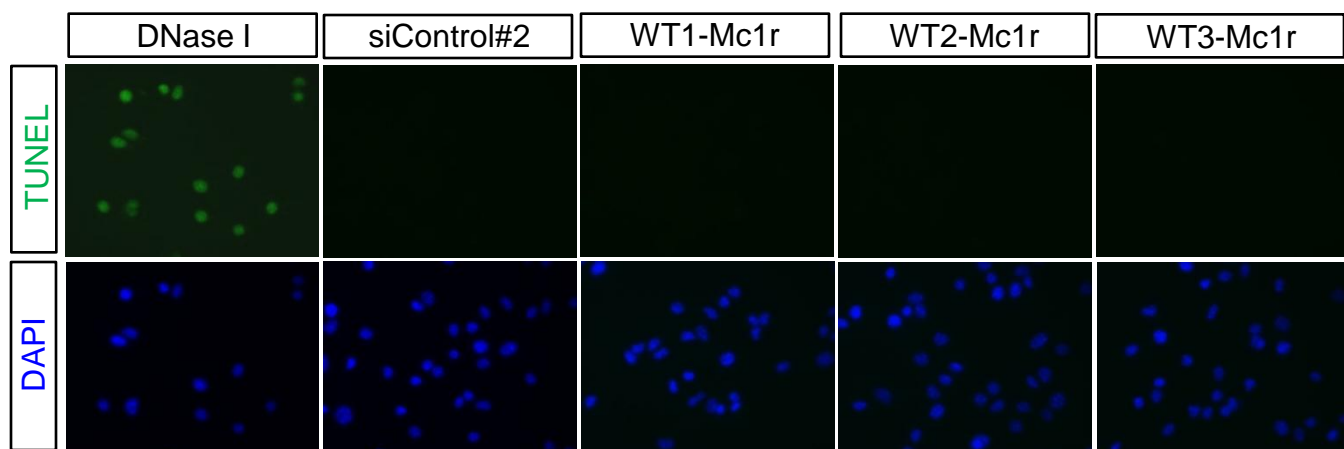

D

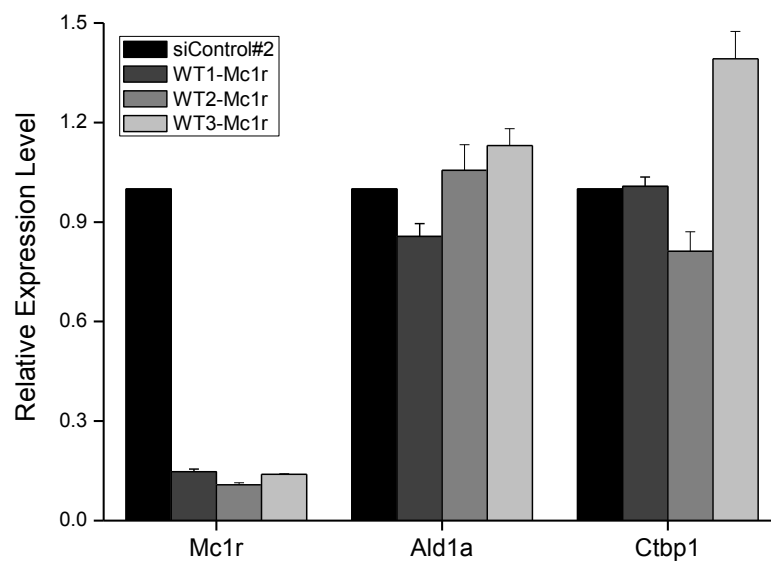

Supplement: Figure S1 — TUNEL assay. B16F10 cells were transfected with indicated siRNAs for 24 hours and replated as in the transwell migration assay. TUNEL assay was performed using TUNEL-Enzyme and TUNEL-Label (Roche). DNase I treated cells were used as positive controls for TUNEL staining. Cells were also stained with specific antibodies to confirm down-regulation of Sox10 (A) and MITF (B). For Mc1r (C), RTPCR was performed (D) in duplicates to confirm the down-regulation (see also Fig. 4G and Fig. 5A). Ald1a and Crbp1 are negative controls for siRNA treatment for Mc1r. (PDF) [file pone.0031477.s001.pdf]

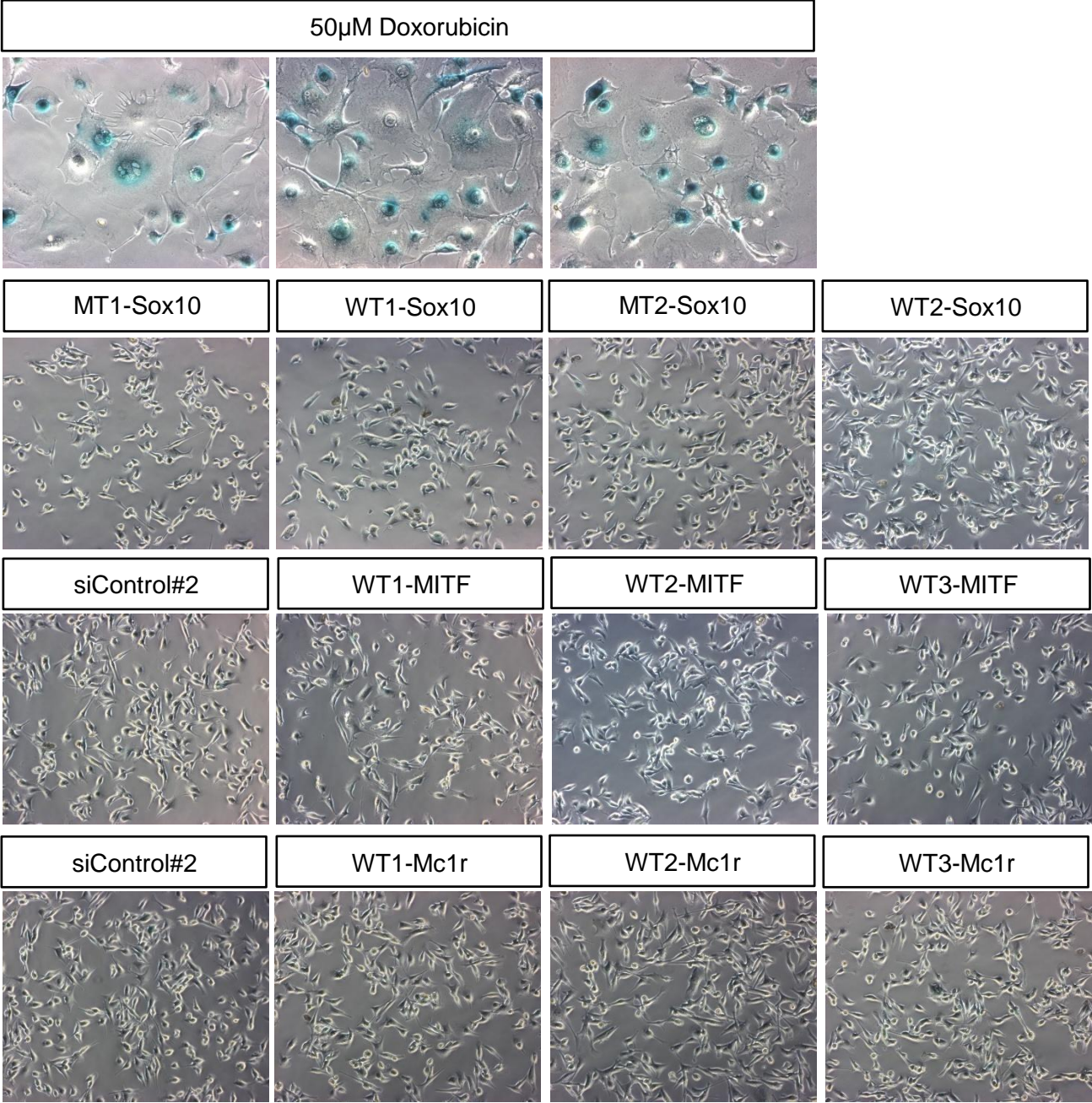

Supplement: Figure S2 — Cell staining for senescence-associated beta-galactosidase activity. B16F10 cells were transfected with indicated siRNAs for 24 hours and replated as in the transwell migration assay. For the positive control, B16F10 cells treated with 50 µM doxorubicin for 5 days were used. Typical X-gal staining and morphological change during senescence are seen only in doxorubicin treated cells. (PDF) [file pone.0031477.s002.pdf]

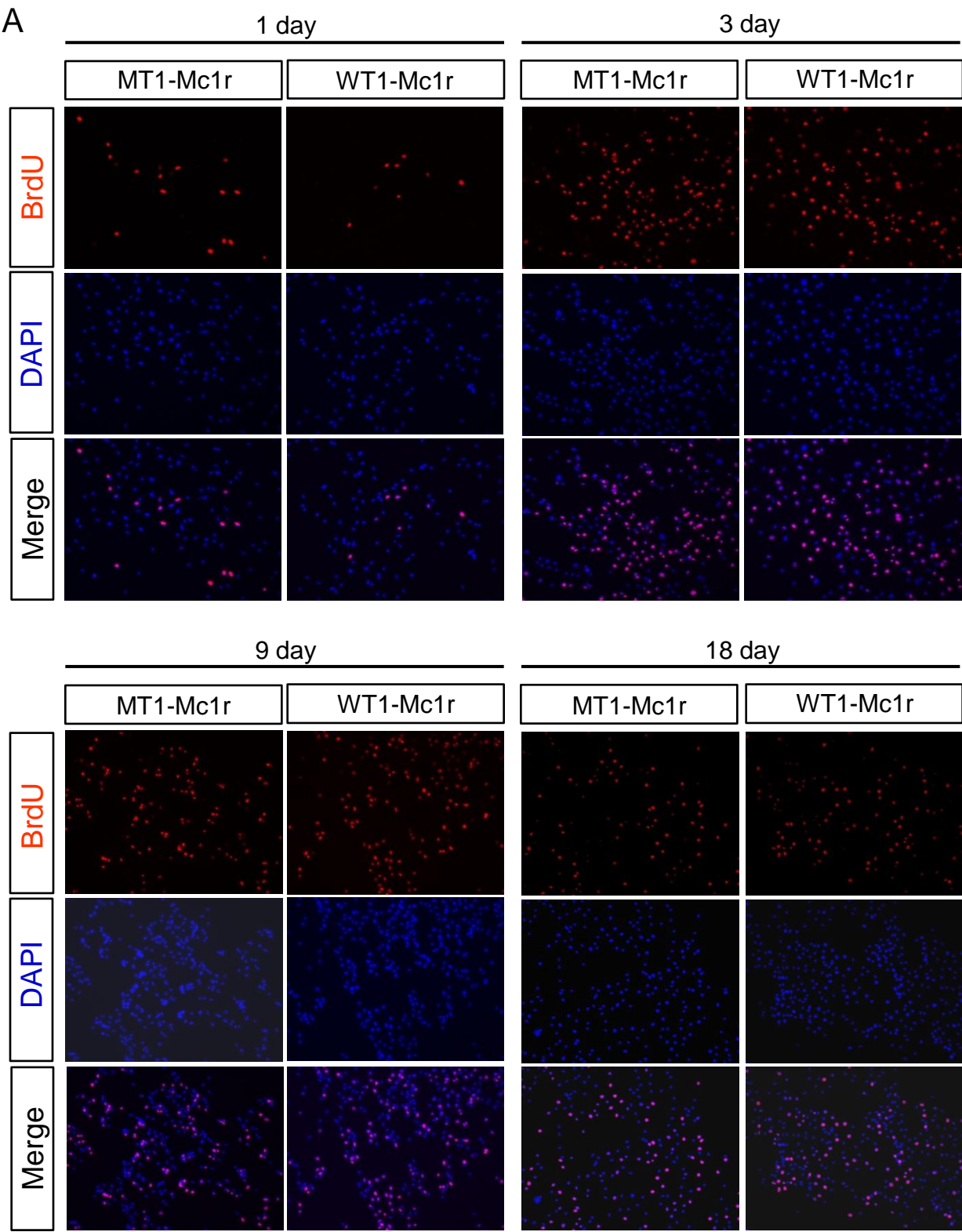

**B**

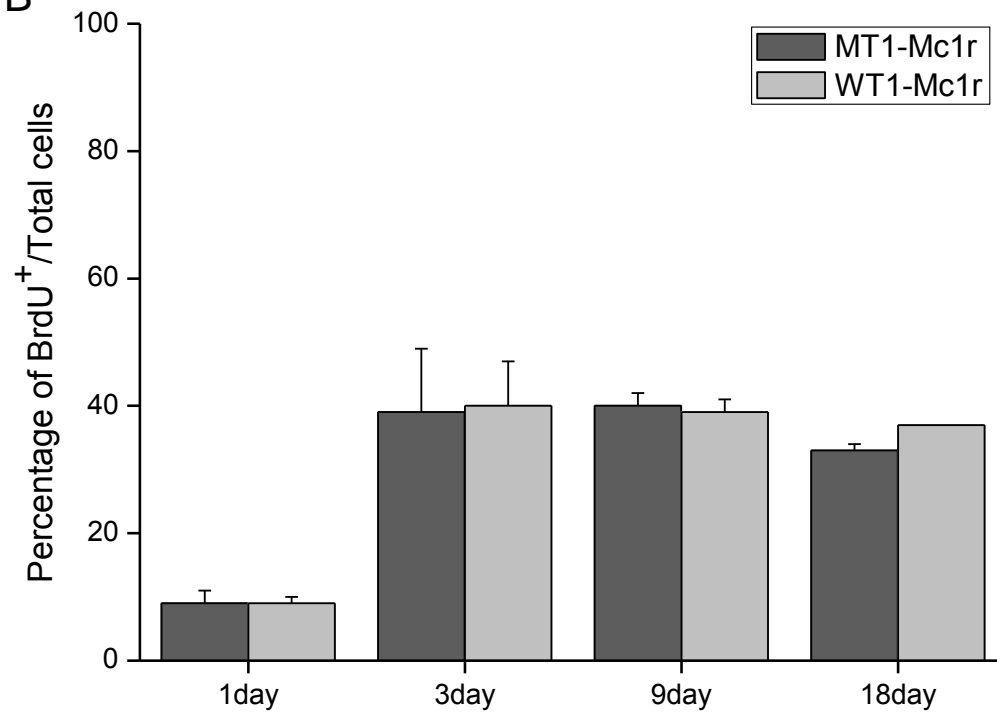

Supplement: Figure S3 — BrdU labeling assay. B16F10 cells were transfected with MT1-Mc1r or WT1-Mc1r and labeled with BrdU for 4 hours at indicated days post transfection. Cells were immunostained for BrdU (A), and the percentages of positive nuclei were determined after DAPI staining. No difference between the two cell populations was observed. (B) Values represent the average of two independent trials, and error bars indicate standard deviation (SD). (PDF) [file pone.0031477.s003.pdf]

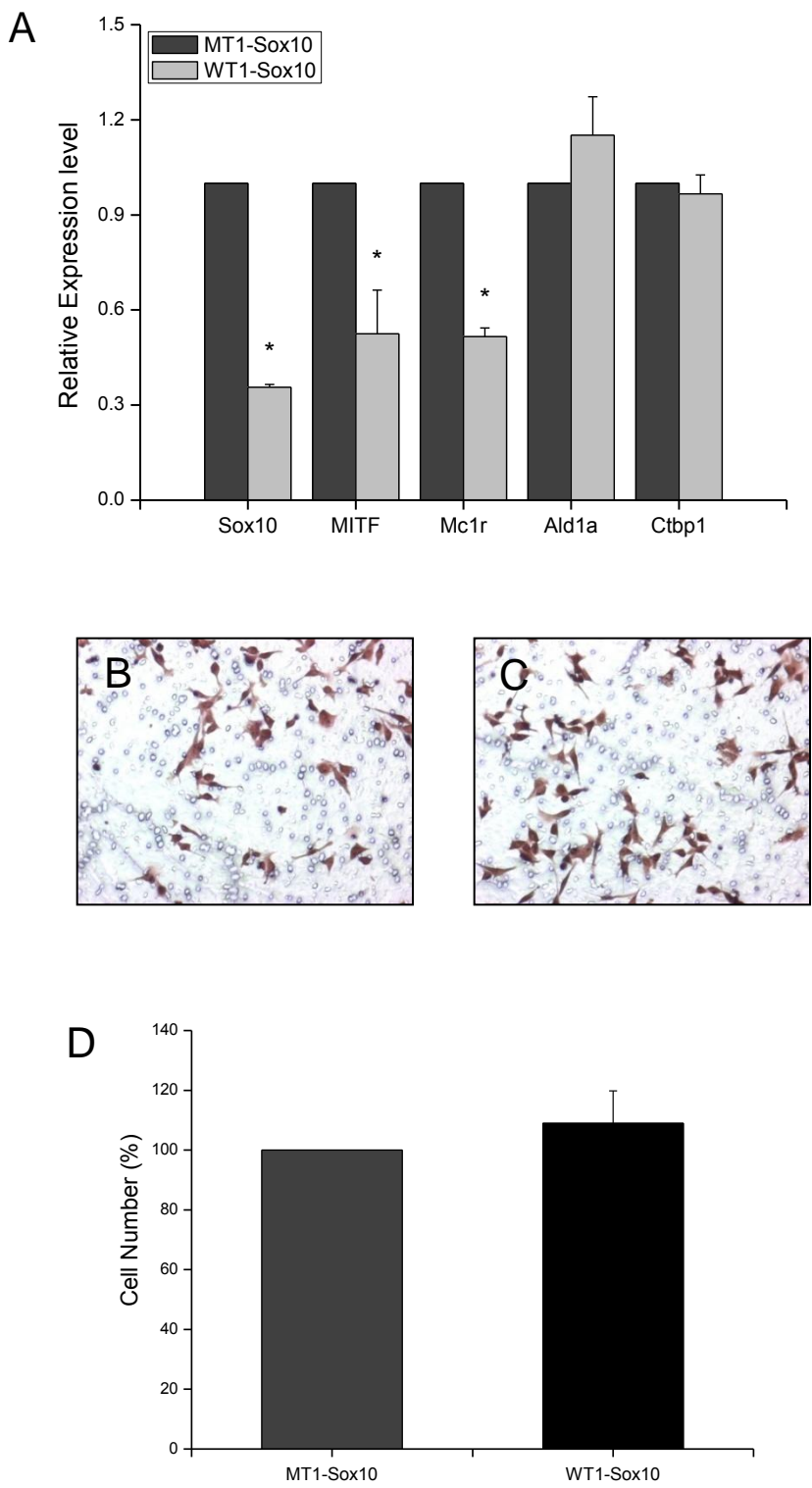

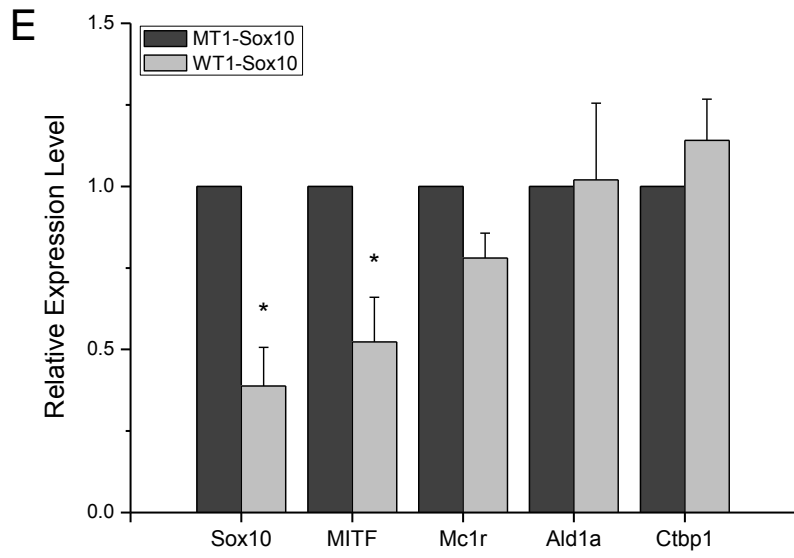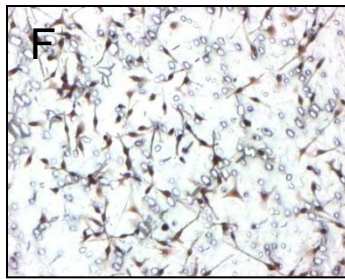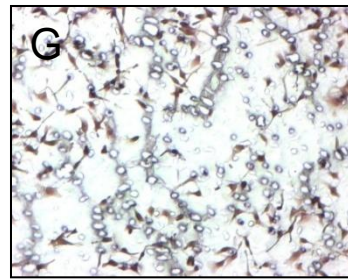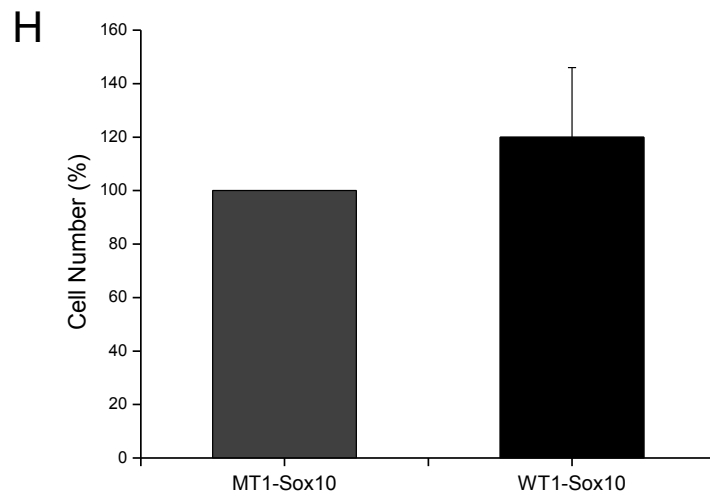

Supplement: Figure S4 — Effect of Sox10 down-regulation on migration of Cloudman S91 and Melan-a melanoma cells. (A) Quantitative real time RTPCR assays were carried out using Cloudman S91 cells transfected with the MT1 or WT1 Sox10 siRNA. Values represent the average of three independent real-time PCR experiments each carried out in duplicates, and error bars represent standard deviations. The asterisk (*) represents a significant difference with the p value of <0.05. Cloudman S91 cells were treated with the MT1 (B) or WT1 (C) Sox10 siRNA and subjected to transwell migration assay. (D) The Graph represents quantitation of transwell migration assay. The effect of Sox10 knockdown on the number of cells that migrated through the filter pores is shown in percentile relative to the control case. Values represent the average of 4 independent trials, and error bars represent standard deviations. (E) Quantitative real time RTPCR assays were carried out using Melan-a cells transfected with the MT1 or WT1 Sox10 siRNA. Values represent the average of three independent real-time PCR experiments each carried out in duplicates, and error bars represent standard deviations. The asterisk (*) represents a significant difference with the p value of <0.05. Melan-a cells were treated with the MT1 (F) or WT1 (G) Sox10 siRNA and subjected to transwell migration assay. (H) The Graph represents quantitation of transwell migration assay. The effect of Sox10 knockdown on the number of cells that migrated through the filter pores is shown in percentile relative to the control case. Values represent the average of 4 independent trials, and error bars represent standard deviations. (PDF) [file pone.0031477.s004.pdf]

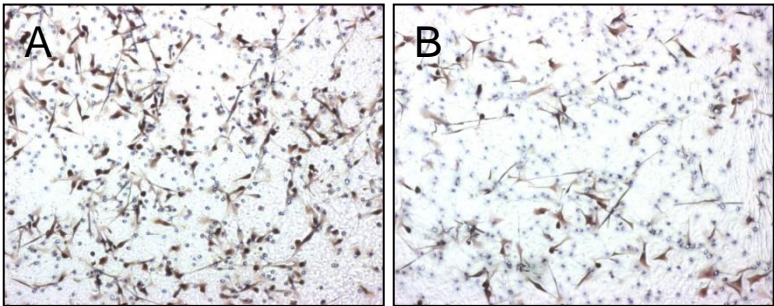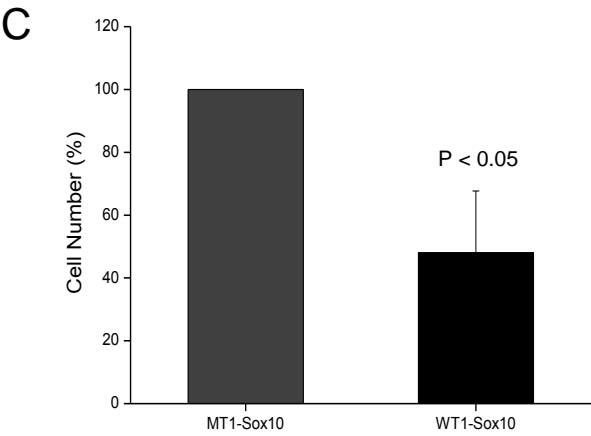

Supplement: Figure S5 — Chemokinetic transwell migration assay. B16F10 cells were transfected with MT1-Sox10 (A) or WT1-Sox10 (B) and subjected to transwell assays with identical media in upper and lower chambers to assess chemokinetic migration. Migration is inhibited in WT1-Sox10 transfected cells. (C) Values represent the average of 5 independent trials, and error bars represent standard deviations. (PDF) [file pone.0031477.s005.pdf]
