# Supplementary material for: Sox10 Controls Migration of B16F10 Melanoma Cells through Multiple Regulatory Target Genes
Source: PLoS One. 2012 Feb 21;7(2):e31477. doi: 10.1371/journal.pone.0031477 (PMC3283624; doi:10.1371/journal.pone.0031477)
Supplement: Methods S1 — Supplementary materials and methods. More information on the TUNEL Assay, Senescence-associated beta-galactosidase activity assay, and BrdU labeling. (DOC) [file pone.0031477.s006.doc]

**Supplementary Materials and Methods**

**TUNEL Assay**

TUNEL assay was performed with TUNEL-Enzyme and TUNEL-Label (Roche) following the manufacturer’s protocol. Briefly, 24hours after siRNA transfection, cells were harvested and replated. Cells were subsequently fixed for 1 hour in freshly prepared 4% paraformaldehyde and incubated with permeabilization solution for 2 min on ice. TUNEL reaction mixture was added to the sample and cells were incubated for 1 hour at 37℃ in a humidified atmosphere in the dark. As the positive control, permeabilized cells treated with DNase I (3U/ml in 50mM Tris-HCl, pH7.5, 1mg/ml BSA) were used. All cells were also counter-stained with DAPI to visualize nuclei.

**Senescence-associated beta-galactosidase activity assay**

Cellular senescence was examined using Senscence Cells Histochemical Staining Kit (Sigma) following the manufacturer’s protocols. Briefly, 24 hours siRNA transfection, cells were harvested and replated. Cells were fixed and incubated with the chromogenic β-gal substrate X-gal in the supplied staining solution. Cells were subsequently washed with PBS and viewed by bright field/phase contrast microscopy. As the positive control, B16F10 melanoma cells treated with 50 μM Doxorubicin for 5 days were used.

**BrdU labeling**

For BrdU labeling, cells were incubated with 10μM BrdU (Sigma) in culture medium for 4h at 37℃ which was followed by fixation and denaturation of DNA with 2N HCl. Cells were subsequently stained with anti-BrdU antibody (DSHB) and Alexa 488 conjugated secondary antibody (Molecular Probes). BrdU-positive and total cells (stained with DAPI) were counted in 3 randomly chosen independent fields.
