# Supplementary material for: Sox10 Controls Migration of B16F10 Melanoma Cells through Multiple Regulatory Target Genes
Source: PLoS One. 2012 Feb 21;7(2):e31477. doi: 10.1371/journal.pone.0031477 (PMC3283624; doi:10.1371/journal.pone.0031477)
Supplement: Table S1 — List and target sequence of siRNAs used for RNA interference assay. (DOC) [file pone.0031477.s007.doc]

**Table S1. List and target sequence of siRNAs used for RNA interference assays.**

| **Gene** | **siRNA target sequence** |
| --- | --- |
| siControl#2 | target firefly luciferase mRNA (U47296) |
| MT1-Sox10 | 5'-GCUGAAGUAGGUACACCAG-3' |
| WT1-Sox10 | 5'-GGUCAAGAAGGAACAGCAG-3' |
| MT2-Sox10 | 5'-UGAAUGCGCUAGAUCAGUA-3' |
| WT2-Sox10 | 5'-UGACUGAGCUGGACCAAUA-3' |
| WT1-Mitf | 5'-CAGUAUGAGCGCAGAAGAA-3' |
| WT2-Mitf | 5'-GCAGAUGGAUGAUGUAAUU-3' |
| WT3-Mitf | 5'-AAACGGAGCAUGCGUGUUA-3' |
| MT1-Mc1r | 5'-GUGAUGAAGCCUGCUACCA-3' |
| WT1-Mc1r | 5'-GUGCUGGAGACUACUAUCA-3' |
| WT2-Mc1r | 5'-CCAGAGUGGCUUUGGUGCA-3' |
| WT3-Mc1r | 5'-ACUCAUGGCGAUUCUGUAU-3' |
| Hyal1 | 5‘-CCAAGGCUCUAACAUGACA-3' |
| Lims2 | 5‘-UGAAGCUCACUCUAAAGAA-3' |
| P2ry2 | 5‘-GGAUACAAGUGUCGUUUCA-3' |
| Tm7sf1 | 5‘-UCACGCUCAUGAACUUGUA-3' |
| Tspan10 | 5‘-GAAUGUGGCUGGACAAGUA-3' |

＊A4 type, dTdT overhang(Dharmacon CO.)
