# Supplementary material for: Sox10 Controls Migration of B16F10 Melanoma Cells through Multiple Regulatory Target Genes
Source: PLoS One. 2012 Feb 21;7(2):e31477. doi: 10.1371/journal.pone.0031477 (PMC3283624; doi:10.1371/journal.pone.0031477)
Supplement: Table S2 — List and sequence of oligonucleotide primers used for real time RTPCR. (DOC) [file pone.0031477.s008.doc]

**Table S2.** **List and sequence of oligonucleotide primers used for real time RTPCR**

| **Gene** | **Sequence** | |
| --- | --- | --- |
| Sox10 | S | 5'-GGACTACAAGTACCAACCTCGG-3' |
|  | AS | 5'-GGACTGCAGCTCTGTCTTTGG-3' |
| Mitf | S | 5’-AACAAGGGAACCATTCTCAAGG-3’ |
|  | AS | 5’-AGATCAGGCGAGCAGAGACC-3’ |
| Hyal1 | S | 5'-CGTAATGCCCTACGTCCAGATC-3' |
|  | AS | 5'-TGGTCACGTTCACGATGAAGG-3' |
| Lims2 | S | 5'-TGCAGTCATGTGATCGAGGGT-3' |
|  | AS | 5'-TTCATAGCACCTCTTACACACGG-3' |
| Mc1r | S | 5'-CTTCTGCCTCAAGGGTGCTG-3' |
|  | AS | 5'-TCAACAGTGGAGCTGAGGACG-3‘ |
| P2ry2 | S | 5'-GTGGACTCTGTCCGTCTTGAGTC-3' |
|  | AS | 5'-CGGTACTCTACTTCCTGGCAGG-3' |
| Tm7sf1 | S | 5'-TACCAATCCTGGGATGGTCC-3' |
|  | AS | 5'-TGCTTGTGCCAGGAAGCTG-3' |
| Tspan10 | S | 5'-TGCAGCCATCATTCACTACTGG-3' |
|  | AS | 5'-TGATGCAGCAAGAGGCTGG-3' |
| Ald1a | S | 5'-GCACTTCGTCGCACAGTGC-3' |
|  | AS | 5'-TGCCTTCAGGTTCTCCTTCTTCC-3' |
| Ctbp1 | S | 5'-GACCTGCTCTTCCACAGTGAC-3' |
|  | AS | 5'-CAGTGCCTTCTCATCCACCAG-3' |
